# Safety and Effectiveness of Meatal Mobilization (MEMO) Technique for Glandular, Coronal, and Subcoronal Hypospadias Repair in Children: A 5-Year Single-Center Study with 105 Hypospadias

**DOI:** 10.3390/biomedicines12040831

**Published:** 2024-04-09

**Authors:** Zenon Pogorelić, Boris Milovac, Tin Čohadžić, Jakov Todorić

**Affiliations:** 1 Department of Surgery, School of Medicine, University of Split, 21000 Split, Croatia; 2 Department of Pediatric Surgery, University Hospital of Split, 21000 Split, Croatia

**Keywords:** hypospadias, distal hypospadias, meatal mobilization, MEMO, Snodgrass, children

## Abstract

Background: This study aims to compare outcomes of treatment, in terms of early and late complications, between the Snodgrass and meatal mobilization (MEMO) techniques in children operated on because of distal hypospadias. Methods: The medical records of 127 children who underwent glandular, coronal, or subcoronal hypospadias repair between 1 January 2019 and 31 December 2023 were retrospectively reviewed. A total of 105 children met the inclusion criteria and were included in further analysis. The inclusion criteria were pediatric patients who underwent glandular, coronal, or subcoronal hypospadias repair using MEMO (*n* = 49) or the Snodgrass technique (*n* = 56) as a comparative group. The primary outcome of this study was the incidence of early and late complications with two different surgical techniques. Secondary outcomes were the duration of surgery, the length of hospital stay, the number of readmissions or unplanned returns to the operating room, and repeat surgeries between groups. Results: The median age of all patients was 17 (interquartile range, IQR 13, 29) months, with a median follow-up of 26 (IQR 17, 34) months. Regarding the type of hypospadias, the majority of patients in both groups were categorized as coronal and subroronal hypospadias. Regarding the incidence of postoperative complications, a significantly lower incidence of postoperative complications was found in the MEMO group compared to the Snodgrass group (*n* = 4; 8.2% vs. *n* = 14; 25%; *p* = 0.037). An urethrocutaneous fistula was the most common complication in the Snodgrass group (*n* = 8; 14.3%), while in the MEMO group, only one patient (2%) developed a fistula (*p* = 0.034). The incidence of meatal stenosis (*p* = 0.621) and wound infections (*p* > 0.999) was low in both groups. No further complications were recorded during the follow-up period. Duration of surgery (41 min (IQR 38, 47) vs. 51 min (IQR 45.5, 61); *p* < 0.001), duration of hospitalization (1 day (IQR 1, 2) vs. 3 days (IQR 2, 6); *p* < 0.001), and time to catheter removal (3 days (IQR 2.5, 5) vs. 6 days (IQR 6, 8); *p* < 0.001) were significantly lower in patients operated on with MEMO compared to the Snodgrass technique. Only one case of readmission due to severe wound infection which led to suturing line dehiscence was recorded in the Snodgrass group. The incidence of redo surgery was significantly lower in the MEMO group than in the Snodgrass group (*n* = 3; 6.1% vs. *n* = 11; 19.6%; *p* = 0.048). Conclusions: MEMO is a safe and effective technique that can be used for the treatment of distal hypospadias. It showed excellent outcomes, cosmetic results, and a low incidence of complications as well as a significantly shorter duration of surgery compared to the Snodgrass technique.

## 1. Introduction

Hypospadias, a congenital anomaly that affects male newborns, is a major challenge for pediatric urology. It is an incomplete virilization of the genital tubercle leading to insufficient development of the tissue along the ventral aspect of the penis [1]. This developmental anomaly occurs during the critical period of embryogenesis, typically between the 7th and 14th week of gestation. At this stage, incomplete fusion of the urethral folds leads to displacement of the urethral meatus, which can be observed along the ventral shaft of the penis, the scrotum, or the perineum [2].

The prevalence of hypospadias is considerable: the incidence rate is estimated to be about 1 in 250 male newborns, which corresponds to approximately 1 in 125 live male births [3]. However, it is crucial to note that hypospadias occurs in a spectrum of severity, ranging from mild to severe cases. Despite decades of research, the exact etiology of hypospadias remains largely unknown. According to current knowledge, a multifactorial cause is suspected, which includes genetic predispositions, hormonal influences, and environmental factors [4,5].

The treatment of hypospadias usually involves surgical intervention to achieve good cosmetic and functional outcome. The timing of surgery is crucial, with recommendations generally ranging from 6 to 18 months. Early intervention aims to optimize results while minimizing potential psychological effects on the child’s growing-up years. Long-term follow-up has shown that the results are generally positive in terms of both cosmetic appearance and sexual function, although there are individual differences [1].

The clinical assessment of hypospadias reveals characteristic features that indicate underlying anatomical abnormalities. Tissue hypoplasia on the ventral side of the penis manifests as a triangular defect often associated with the division of the fan-shaped corpus spongiosum. This division, which is typically observed proximal to the ectopic meatus, can be demarcated by a small ridge of skin on the ventral skin. Associated anomalies often include an ectopic urethral meatus, ventral penile curvature (chordee), and defects in the ventral foreskin [6]. Advances in genetic research promise further insights into the molecular mechanisms underlying hypospadias. The identification of specific gene mutations involved in penile and testicular development offers potential targets for personalized diagnostic and therapeutic approaches [7,8,9].

The scope of surgical treatment for distal hypospadias ranges from two-stage procedures to single-stage repairs, with popularity and preferences fluctuating over time. For example, Tubularized Incised Plate Repair (TIP), introduced by Snodgrass, was initially recognized for distal hypospadias but later used in complex cases with promising results. The choice of surgical technique is often determined by intraoperative assessment, taking into account factors such as the position of the meatus, penile curvature, and the condition of the ventral skin before and after degloving [10].

In recent years, the technique of Meatal Mobilization with Glanuloplasty Inclusive (MMGPI) has emerged as a modification of MAGPI (Meatal Advancement and Glanuloplasty Inclusive) with the aim of further improving surgical outcomes in the repair of distal hypospadias. This technique requires the careful assessment of penile anatomy, dorsal foreskin, and urethral plate mobility to determine its feasibility [1,2,7,11].

Complications of hypospadias surgery remain a concern, including problems such as urethrocutaneous fistulas, meatal stenosis, urethral strictures, wound infections, and others. The prevalence of these complications depends on factors such as surgical technique, patient anatomy, and postoperative care [12].

The Snodgrass technique is a widely used one-stage repair of distal hypospadias that aims to create a neourethra from the urethral plate. In this technique, the incised urethral plate is tubularized to create a neourethra, followed by a glanuloplasty to reconstruct the glans penis. The Snodgrass technique has gained popularity due to its simplicity, low complication rate, and favorable cosmetic results. Because the natural urethral plate is preserved and no tissue grafts are required, the Snodgrass technique offers advantages in terms of operative time, postoperative recovery, and overall patient satisfaction [13].

Specifically, the Snodgrass technique begins with degloving the penis to expose the urethral plate. The urethral plate is then incised along the midline and the edges are sutured together to form a tube, creating the neourethra. Care is taken to ensure that the neourethra is sufficiently wide and deep to avoid strictures and achieve optimal urinary function. Once tubularization is complete, a glanuloplasty is performed to reconstruct the glans penis and achieve a cosmetically pleasing appearance. The Snodgrass technique is associated with high success rates and low complication rates, making it the preferred choice for many surgeons in the treatment of distal hypospadias [14].

The MEMO (meatal mobilization) technique is a surgical approach that aims to correct distal hypospadias while preserving the native meatus and achieving excellent cosmetic results. In the MEMO technique, the urethral plate is preserved and mobilized to increase the length of the urethra, allowing for a more natural position of the meatus. This technique minimizes the risk of regression or narrowing of the meatus that often occurs with conventional procedures. By preserving the natural meatus and the integrity of the urethral plate, MEMO offers the advantage of an improved urinary stream and a lower risk of complications such as fistula formation [15].

Objective parameters and validated scoring systems help evaluate factors like cosmetic appearance, urinary function, and complication rates comprehensively. Techniques like MEMO have shown promising results in long-term follow-up, offering low complication rates and excellent cosmetic outcomes, especially in distal hypospadias cases [16].

This study aims to compare outcomes of treatment, in terms of early and late complications, between the Snodgrass and MEMO techniques in children operated on because of distal hypospadias in our institution.

## 2. Methodology

### 2.1. Patients

**Figure 1:**
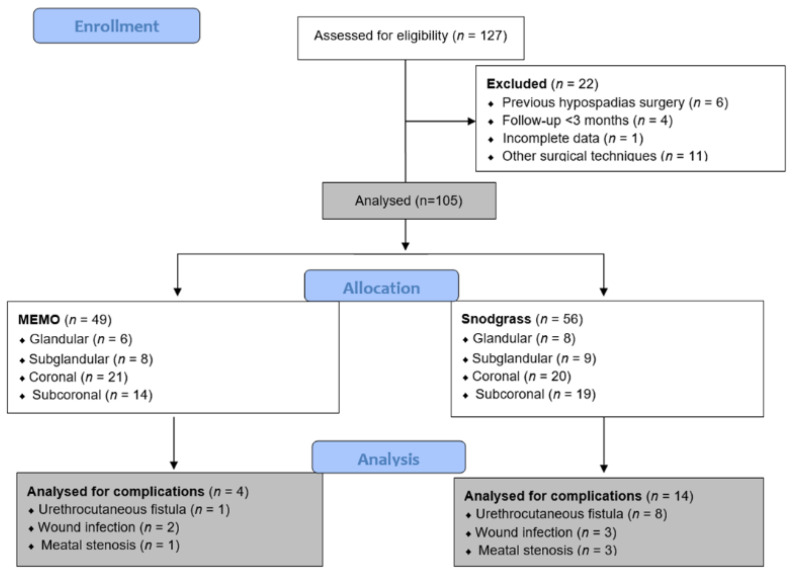
Flow chart of the study.

The medical records of 127 pediatric patients who underwent glandular, coronal, or subcoronal hypospadias repair at the Department of Pediatric Surgery, University Hospital of Split, between 1 January 2019 and 31 December 2023 were retrospectively reviewed. A total of 22 children met one or more exclusion criteria and were excluded from the study. Finally, 105 children met the inclusion criteria and were included in further analysis. The inclusion criteria were pediatric patients who underwent glandular, coronal, or subcoronal hypospadias repair using the MEMO or Snodgrass technique, as a comparative group, in our department by two experienced pediatric urologists (J.T. (*n* = 38; 36.2%) and Z.P. (*n* = 67, 63.8%)). Patients who were older than 17 years, patients who previously received hypospadias repair surgery, patients who had undergone other surgical techniques for distal hypospadias repair, patients with significant comorbidities, patients with less than 3 months of follow-up, or patients with incomplete data in medical records were excluded from the study. The flow diagram of the study is shown in Figure 1.

### 2.2. Ethical Aspects

The study was conducted in accordance with the Declaration of Helsinki of the World Medical Association, and the Institutional Review Board of our hospital approved the study (approval number: 500-03/23-01/219; date of approval: 27 November 2023).

### 2.3. Outcomes of the Study

The primary outcome of this study was the incidence of early and late complications with two different surgical techniques for glandular, coronal, and subcoronal hypospadias repair. Secondary outcomes were the duration of surgery, the length of hospital stay, the number of readmissions within 30 days after surgery (ReAd) [17], unplanned returns to the operating room (uRORs) [18], and redo surgeries between groups.

### 2.4. Data Collection and Study Design

Depending on the surgical technique used for hypospadias repair, the children were divided into two study groups. The patients from first group had undergone the MEMO technique (*n* = 49) for hypospadias repair [16]. Grafted TIP urethroplasty developed by Warren Snodgrass was used in the second group of patients (*n* = 56) [19]. The patient baseline demographics data (age, weight, height, body mass index), type of hypospadias (glandular, subglandular, coronal, subcorneal), and preoperative urinary symptoms (meatal stenosis, dripping, or straining) were compared between the study groups. The groups were also compared in terms of early (urethrocutaneous fistula formation, wound infection, dehiscence of suture line, necrosis of the skin) and late postoperative complications (meatal stenosis, residual chordee, urethral stricture, scar, urinary symptoms), the duration of surgery, the time of catheter removal, the length of hospital stay, repeat surgery, ReAd, and uRORs.

### 2.5. Suturgical Techniques

#### 2.5.1. MEMO Technique

**Figure 2:**
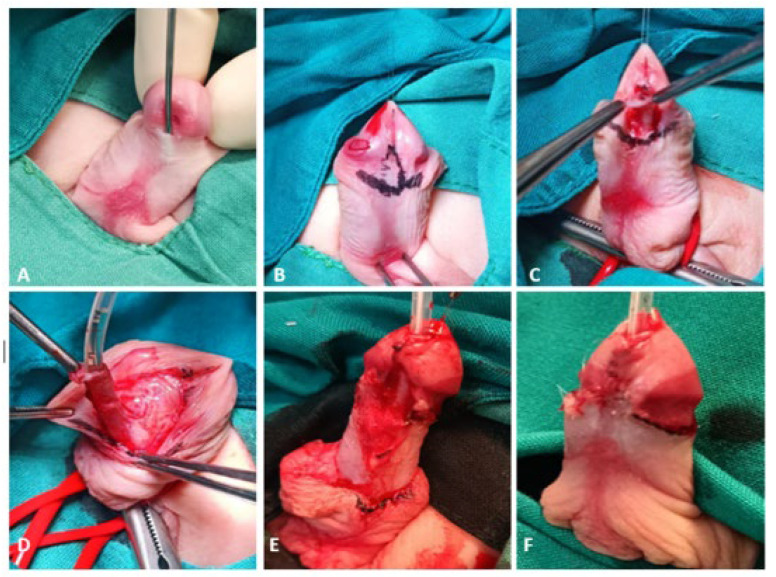
MEMO technique: (**A**)—Coronal hypospadias in a 16-month-old pediatric patient; (**B**)—the holding sutures in place and the marked incision line; (**C**)—meatal incision and verification of the mobility of the ventral side of the urethra; (**D**)—degloving of the penile skin and mobilization of the urethra; (**E**)—mobilization of the meatus to a tip of the glans and glanduloplasty; (**F**)—final result.

All patients underwent surgery for glandular, coronal, or subcoronal hypospadias (Figure 2A) under general anesthesia in the supine position. A glandular stay suture is placed and a silicone urethral catheter is inserted to preserve the ventral urethral mucosa during penile skin dissection. A bandage is applied to the root of the penis to prevent bleeding during the procedure. An incision line is marked subcoronally on the inner prepuce (Figure 2B). The mobility of the ventral side of the urethra is checked with forceps. Suppose the surgeon determines that the urethra can be easily mobilized and MEMO is feasible. In that case, the procedure is continued (Figure 2C). After the circular incision is made, the penile skin is dissected downward as far as necessary for dissecting chordee tissue. After the penile skin is dissected, the meatus is freed circumferentially with sharp scissors, starting laterally on both sides of the meatus, and the urethra is mobilized (Figure 2D). The mobilization plane dorsally corresponds to the fascia albuginea of the corpora. The length of mobilization depends on the achieved mobility of the urethra. After the urethra has been mobilized, the spongiosal tissue of the glans should be mobilized laterally to prepare the glanular wings for ventral glanuloplasy. The incision of the glans is made ventrally to the tip of the glans. Excess glandular mucosa is resected on both medial sides of the glandular wings. The neomeatus is created by mobilizing the meatus at the tip of the glans (Figure 2E). The neomeatus is adapted to the glans with single 6/0 triclosan-coated polydioxanone sutures (PDS Plus^®^ 6/0, Ethicon, Johnson & Johnson, Diegem, Belgium). Suturing of the glandular epithelium is followed by reconstruction of the ventral internal foreskin. A resection of the residual skin of the dorsal prepuce is performed to its final limit, and the inner prepuce is sutured to the skin (Figure 2F). If there is a ventral skin deficit for penile shaft reconstruction, the dorsal skin portions should be rotated ventrally. Before applying the wound dressing, 1% lidocaine (Lidokain, Belupo, Koprivnica, Croatia) is applied subcutaneously. The standard wound dressing consists of Vaseline gauze, silver sulfadiazine cream (Dermazin, Salutas Pharma GmbH, Osterweddingen, Germany), and COBAN™ self-adherent wrap (3M™, Neuss, Germany). Depending on local findings and the operating surgeon’s preferences, the urethral catheter and dressing are removed 2–5 days after the procedure.

#### 2.5.2. Snodgrass Technique

All patients underwent surgery for glandular, coronal, or subcoronal hypospadias under general anesthesia in the supine position. In this procedure, glandular stay suture is placed and a silicone urethral catheter is inserted to preserve the ventral urethral mucosa during penile skin dissection. A bandage is applied to the root of the penis to prevent bleeding during the procedure. A skin incision is performed about 2–3 mm proximal to the meatus of the urethra with a flap dissection of the urethral plate, starting from the meatus to the upper end of the glans. After that, a circular skin incision along the coronal sulcus is performed. Degloving of penile skin is performed to the base of the penis. An erection test with 0.9% NaCl is performed to determine penile curvature. Then, the urethral plate is incised in the midline from the meatus to the top of the glans. A continuous, absorbable, triclosan-coated polydioxanone suture (PDS Plus^®^ 6/0, Ethicon, Johnson & Johnson, Diegem, Belgium), which is not tensioned, is used to form the neourethra over the urethral catheter. The suture line starts at the proximal end and ends at the neomeatus. Several single-loop sutures are applied to the neourethra to secure the suture line. The neourethra is covered with a well-vascularized internal prepuce flap graft, which is mobilized on the ventral surface and secured with single sutures. The reconstruction of the glans is performed with the same absorbable single sutures. The excess foreskin is excised after skin reconstruction and suturing of the inner foreskin to the skin. Before applying the wound dressing, 1% lidocaine (Lidokain, Belupo, Koprivnica, Croatia) is applied subcutaneously. The standard wound dressing consists of Vaseline gauze, silver sulfadiazine cream (Dermazin, Salutas Pharma GmbH, Osterweddingen, Germany), and COBAN™ self-adherent wrap (3M™, Neuss, Germany). Depending on local findings and the operating surgeon’s preferences, the urethral catheter is removed 7–10 days after the procedure.

### 2.6. Postoperative Protocol and Follow-Up

**Figure 3:**
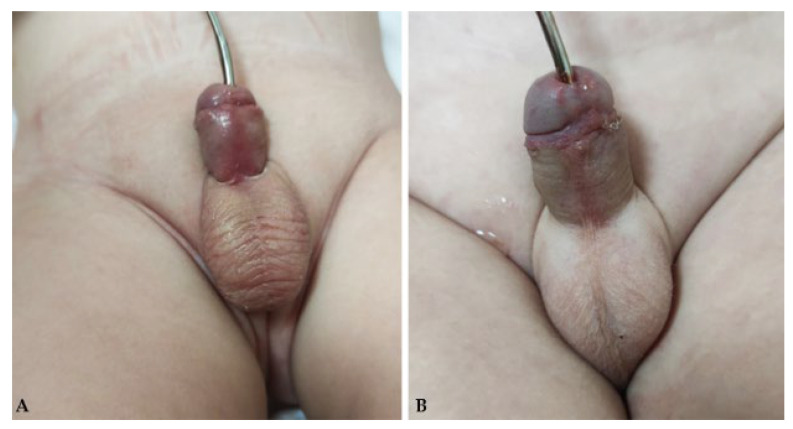
One-month postoperative follow-up after MEMO technique: (**A**)—A 16-month-old pediatric patient with coronal hypospadias. (**B**)—A 24-month-old pediatric patient with subcoronary hypospadias. In both cases, an excellent cosmetic effect is seen, with a well-positioned meatus without signs of stenosis. The penis shows no significant curvature.

In this study, in most cases, the wound dressing was changed on the third and seventh postoperative days. In cases where the local status required it, the dressing was changed even more frequently. For pain relief, paracetamol (Perfalgan, Bristol-Myers Squibb S.r.l., Agen, France) was administered at a dose of 10–15 mg/kg or ibuprofen (Brufen, Mylan, Zagreb, Croatia) at a dose of 10 mg/kg. In most cases, cephalexin (Cefalexin, Belupo, Koprivnica, Croatia) at a dose of 25–50 mg/kg or gentamicin (Gentamicin, Belupo, Koprivnica, Croatia) at a dose of 3–5 mg/kg was used for antibiotic prophylaxis until the urinary catheter was removed. In previous years, the patients were discharged after the removal of the urinary catheter, while recently, in most of the cases, the patients were discharged on the first or second postoperative day. After discharge, patients were followed up in our outpatient clinic to detect complications. Visits were scheduled for the 7th to 14th day after surgery, depending on the type of hypospadias and the surgeon’s preference. The late follow-up program consisted of a physical examination and, if necessary, dilatation of the urethra (Figure 3). Visits were scheduled for 1, 3, 6 and 12 months after the operation to assess the presence of late complications.

### 2.7. Statistical Analysis

Statistical Package for the Social Sciences—SPSS 28.0 (IBM Corp, Armonk, NY, USA) and Microsoft Excel for Windows version 11.0 (Microsoft Corporation, Redmond, WA, USA) were used for statistical analysis. Medians and interquartile ranges (IQRs) were used to describe the distribution of quantitative data, whereas categorical data were described with absolute numbers and percentages. For the comparison of continuous variables, the Mann–Whitney U test was applied. Categorical variables were compared using chi-square or two-tailed Fisher’s exact depending to the frequency of events in a given cell. All *p*-values < 0.05 were considered significant.

## 3. Results

### 3.1. Demographic and Clinical Data of the Patients

During the 5-year study period, 105 children who underwent surgery for distal hypospadias met the inclusion criteria and were included in the study. The median age of all patients was 17 (IQR 13, 29) months, with a median follow-up of 26 (IQR 17, 34) months. Regarding the type of hypospadias, the majority of patients had coronal or subroronal hypospadias. Lower incidence was found for subglandular and glandular types. No statistically significant differences were found between the groups with regard to demographic characteristics, the type of hypospadias, and preoperative urinary symptoms (Table 1).

### 3.2. Comparison of Surgical Techniques in Relation to Study Outcomes

**Figure 4:**
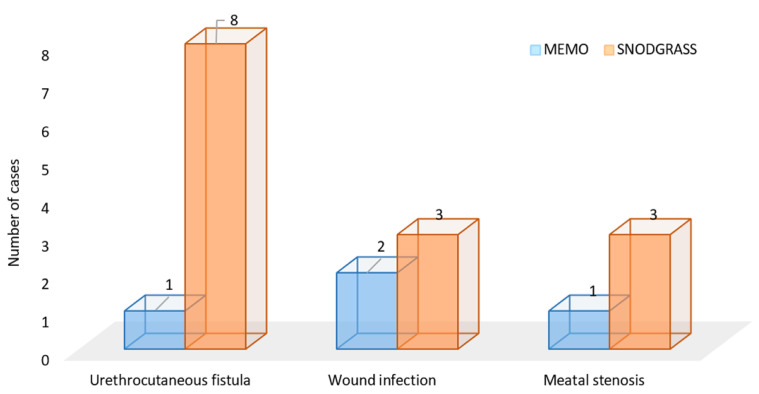
Comparison of postoperative complications between MEMO (*n* = 4) and Snodgrass technique (*n* = 14).

Regarding the main outcome of the study—the incidence of postoperative complications—a significantly lower incidence of postoperative complications was found in the MEMO group compared to the Snodgrass group (*n* = 4; 8.2% vs. *n* = 14 (25%); *p* = 0.037). A urethrocutaneous fistula was the most common complication in the Snodgrass group (*n* = 8; 14.3%), while in the MEMO group, only one patient (2%) developed a fistula (*p* = 0.034). The incidence of meatal stenosis (*p* = 0.621) and wound infections (*p* > 0.999) was low in both groups (Figure 4). No further complications were recorded during the follow-up period.

The duration of surgery (41 min (IQR 38, 47) vs. 51 min (IQR 45.5, 61); *p* < 0.001), duration of hospitalization (1 day (IQR 1, 2) vs. 3 days (IQR 2, 6); *p* < 0.001), and time to catheter removal (3 days (IQR 2.5, 5) vs. 6 days (IQR 6, 8); *p* < 0.001) were significantly lower in patients operated on with MEMO compared to the Snodgrass technique. No cases of uROR occurred in either group. Only one case of ReAd was recorded in the Snodgrass group. The incidence of redo surgery was significantly lower in the MEMO group than in the Snodgrass group (*n* = 3; 6.1% vs. *n* = 11; 19.6%; *p* = 0.048). A comparison of postoperative complications; intra- and postoperative outcomes; and rates of uROR, ReAd, and redo surgery is shown in Table 2.

## 4. Discussion

The main goal that every surgeon wants to achieve in the treatment of hypospadias is a straight penis with full functionality and as normal an appearance as possible. Numerous techniques have been developed, of which the TIP (Snodgrass technique) is the method of choice for the distal type of hypospadias. The MAGPI method with numerous modifications, including the MEMO technique, has proven to be very effective. Because there are not many studies comparing MEMO and Snodgrass, our study aimed to compare the treatment outcomes when using these techniques in the treatment of patients with distal hypospadias. This study demonstrated a statistically significant lower number of postoperative complications with the MEMO technique compared to the Snodgrass technique. In addition, the MEMO technique showed a significantly lower number of urethrocutaneous fistulas when the individual complications were considered separately. The occurrence of infections or stenosis had a low incidence in both techniques. A shorter duration of surgery and a shorter length of hospital stay were also observed when the MEMO technique was used. The MEMO technique showed faster catheter removal compared to the Snodgrass technique. A statistically significantly lower number of reoperations after performing the MEMO technique was observed, as well.

As hypospadias can have a wide range of severity, more than 250 different techniques have been described to date. A review of the literature shows that TIP, first described by Snodgrass, is still the most commonly used technique in the treatment of distal hypospadias, with MAGPI, hybrid Mathieu urethroplasty, onlay preputial flap, dorsal inlay graft urethroplasty, and many others mentioned as alternatives [20]. A meta-analysis conducted by Zhang et al., which included 16 studies and compared the postoperative outcomes of 1386 patients operated on with the Snodgrass or Mathieu technique, showed an equal incidence of fistulas and wound dehiscence but a higher frequency of meatal stenoses in patients operated on using the Snodgrass technique [21]. The same results were observed in another meta-analysis by Winberg et al. Their results show an incidence of 13% for fistulas and 5% for meatal stenosis, which is very similar to the results obtained in our study when performing the Snodgrass technique [22]. In a study by El-Helaly et al., on 66 patients with distal hypospadias, the Snodgrass technique and the transverse preputial onlay flap (TPOF) were compared. The results show that complications such as fistulas occur more frequently after the Snodgrass technique than after TPOF. However, the Snodgrass technique results in a more normal-looking glans and a meatus that resembles a natural vertical slit-shaped meatus [23].

In recent years, methods based on a simple advancement of the meatus rather than a true urethroplasty have been increasingly favored. MAGPI proved to be extremely useful in the reconstruction of distal hypospadias that had not previously been operated on regularly, as the harm was unfavorably compared to the benefit. To improve the results, many modifications have been presented in the literature, and a similar method described by Seibold is MEMO [15]. Some advantages of MEMO compared to MAGPI are the ability to mobilize the meatus and create an anastomosis without pressure, the creation of a slit-like meatus, and the use of one central and two lateral sutures, which places less stress on the meatus. All this helps to reduce meatal retraction and stenosis, which are among the most common complications. Moradi et al., found an incidence of 3.3% for meatal stenosis and 5.8% for meatal retraction in their study of 120 patients [14]. Another study, in which 46 patients were operated on using the MEMO technique, showed that no patient had a urethral stenosis or fistula postoperatively. Only one patient had a meatal retraction. They also point out that it is important for a successful operation that the urethra is not fixed and that the ventral urothelium is not too thin, as in such cases, stretching of the urethra increases the risk of complications. The Snodgrass technique is recommended for such patients [15]. Considering the fact that MEMO and similar techniques are characterized by preserving the integrity of the native meatus and ureteral plate without the need for incision as in the Snodgrass technique, a lower incidence of complications (mainly urethral strictures and fistulas) can be expected. A study by Askarpour et al., compared the modified MAGPI with the Snodgrass technique and found a statistically significantly higher incidence of urethrocutaneous fistulas in patients operated on using the Snodgrass technique [11]. Seibold et al., re-examined 99 patients five years after surgery, none of whom had a stenosis, and only 1 patient developed a fistula postoperatively. The MEMO technique had high individual patient satisfaction and excellent long-term success, confirmed by various objective questionnaires [16].

As previously mentioned, the most common complications with the Snodgrass technique include urethrocutaneous fistulas, meatal stenosis, and urethral strictures [24,25]. According to our results, the most common complication with the Snodgrass technique was urethrocutaneous fistulas, with a frequency of 13%. Buisson et al., came to identical conclusions in their study, with a frequency of fistulas and stenoses of 13% and 4%, respectively. They also stated that they were able to further reduce the incidence of complications by gaining experience and practicing a deeper incision [26]. Pfistermuller et al., included 49 studies in their meta-analysis and showed that performing the Snodgrass technique for proximal hypospadias had a higher rate of all postoperative complications compared to distal hypospadias, which is why it is recommended to consider a staged repair for proximal types [27]. A study by Snodgrass et al., showed that complications of urethroplasty doubled in patients who underwent a second hypospadias urethroplasty. These results show the importance of making a quality assessment of each patient and choosing the best technique to be successful from the first operation [28].

To improve the outcome of surgery, several studies have attempted to establish a correlation between the occurrence of complications and the anatomical characteristics of the patient. A prospective study of 42 patients showed that the cosmetic outcome of hypospadias repair could not be related to the width of the urethral plate (UPW). Still, the functional outcome could be predicted, and it was statistically better in patients with a UPW > 8 mm [29]. In contrast to these conclusions, studies by Galal et al., show that a UPW narrower than 8 mm is not associated with a greater number of complications but with a worse aesthetic outcome after performing the Snodgrass technique [30]. To determine whether the Snodgrass technique is appropriate for patients, Bush and Snodgrass presented the same results and concluded that it is not necessary to measure the width or categorize the plate as long as the incision is made deep into the corpus [31]. Several studies show that the position of the meatus and the size of the glans (less than 14 mm) are important predictive factors for the development of postoperative complications such as fistulas, stenosis, or dehiscence of the glans. Therefore, it is advisable to assess the width of the glans and the position of the meatus preoperatively to predict the risk of complications [32,33,34,35]. The postoperative wound dressing and the duration of catheter removal have also been observed as potential risk factors for complications. Several studies have confirmed that catheter duration is not related to the occurrence of complications, but further research is needed to confirm this [36,37]. In addition, a recently published study has clearly shown that complication rates related to urethrocutaneous fistulas, infections, and wound dehiscence are closely related to the choice of suture material. Polydioxanone sutures (PDSs) are associated with a lower incidence of urethrocutaneous fistulas compared to other suture materials [38]. In addition, triclosan-coated PDSs showed a significantly lower incidence of infection and wound dehiscence compared to uncoated PDSs [39].

The retrospective design of the study is a main limiting factor, as only parameters that have been previously documented were available. Another limitation is the relatively small sample size. A multicenter study with more patients would be more meaningful. The operations were not performed by the same surgeon. If all patients were operated on by the same surgeon, the difference in experience with a particular surgical technique that may exist between two surgeons would be reduced to zero. In addition, the average follow-up time of patients was 26 months, which is relatively short considering that nowadays, it is encouraged to show results after a long-term follow-up of patients. Therefore, future studies with a large number of patients followed up over a longer period and clear criteria are needed to evaluate the best operative intervention for distal hypospadias.

## 5. Conclusions

The study shows that the MEMO technique is associated with excellent outcomes and cosmetic results and a low incidence of complications, especially in terms of urethrocutaneous fistulas. In addition, significantly shorter durations of surgery and frequencies of reoperations were observed when compared to the Snodgrass technique. Due to its simplicity and very good aesthetic result, MEMO can certainly be a surgical alternative in the treatment of distal hypospadias.

## Figures and Tables

**Table 1:** Patient demographics, preoperative voiding dysfunction, and type of hypospadias (*n* = 105).

Variables	Group I (*n* = 49)	Group II (*n* = 56)	*p*
	MEMO	SNODGRASS	
Demographic characteristics of patients; median (IQR) or *n* (%)			
Age (months)	17 (12, 35.5)	17 (13, 29)	0.342 *
Height (cm)	90 (82, 104)	90 (84, 98)	0.818 *
Weight (kg)	13 (11, 15.2)	12 (10, 14)	0.173 *
BMI (kg/m^2^)	16.2 (14.7, 17.5)	15.6 (14.1, 17)	0.190 *
Associated anomalies	6 (12.2)	7 (12.5)	0.940 ^†^
Type of hypospadias; *n* (%)			
Glandular	6 (12.2)	8 (14.3)	0.881 ^†^
Subglandular	8 (16.3)	9 (16.1)	
Coronal	21 (42.9)	20 (35.7)	
Subcoronal	14 (28.6)	19 (33.9)	
Preoperative urinary difficulties; *n* (%)			
Meatal stenosis	1 (2)	1 (1.8)	>0.999 ^‡^
Dripping	0 (0)	1 (1.8)	>0.999 ^‡^
Straining	1 (2)	0 (0)	>0.999 ^‡^

* Mann–Whitney U-test; ^†^ chi-square test; ^‡^ Fisher’s exact test; MEMO—meatal mobilization technique; BMI—body mass index; IQR—interquartile range.

**Table 2:** Treatment outcomes of patients who underwent surgery for hypospadias (*n* = 105).

Variables	Group I (*n* = 49)	Group II (*n* = 56)	*p*
	MEMO	SNODGRASS	
Complications; *n* (%)			
Total number of complications	4 (8.2)	14 (25)	0.037 *
Urethrocutaneous fistula	1 (2)	8 (14.3)	0.034 *
Wound infection	2 (4.1)	3 (4.4)	>0.999 *
Meatal stenosis	1 (2)	3 (4.4)	0.621 *
Intraoperative/postoperative outcomes; median (IQR)			
Duration of surgery (min)	41 (38, 47)	51 (45.5, 61)	<0.001 ^†^
Length of hospital stay (days)	1 (1, 2)	3 (2, 6)	<0.001 ^†^
Catheter removal (days)	3 (2.5, 5)	6 (6, 8)	<0.001 ^†^
ReAd/uROR/redo surgery; *n* (%)			
ReAd	0 (0)	1 (1.8)	>0.999 *
uROR	0 (0)	0 (0)	-
Redo surgery	3 (6.1)	11 (19.6)	0.048 *

* Fisher’s exact test; ^†^ Mann–Whitney U-test; MEMO—meatal mobilization technique; IQR—interquartile range; ReAd—readmission within 30 days after index surgery; uROR—unplanned return to the operating room.

## Data Availability

The data supporting this study’s findings are available upon request from the corresponding author.

## References

[1] Van der Horst H.J., de Wall L.L. (2017). Hypospadias, all there is to know. Eur. J. Pediatr..

[2] Kaefer M. (2021). Hypospadias. Semin. Pediatr. Surg..

[3] Pogorelić Z. (2022). Surgical approach to the treatment of urinary tract anomalies. Liječ Vjesn..

[4] Zhang Z., Zhang Q., Liu Z., Wang C., Chen H., Luo X., Shen L., Long C., Wei G., Liu X. (2024). Rab25 is involved in hypospadias via the β1 integrin/EGFR pathway. Exp. Cell Res..

[5] Arboleda V.A., Sandberg D.E., Vilain E. (2014). DSDs: Genetics, underlying pathologies and psychosexual differentiation. Nat. Rev. Endocrinol..

[6] Catti M., Demède D., Valmalle A.F., Mure P.Y., Hameury F., Mouriquand P. (2008). Management of severe hypospadias. Indian. J. Urol..

[7] Sparks T.N., Society for Maternal-Fetal Medicine (SMFM) (2021). Hypospadias. Am. J. Obstet. Gynecol..

[8] Nordenskjöld A., Lagerstedt-Robinson K., Anderlid B.M., Lundin J. (2023). Tissue specific trisomy 15 mosaicism associated with urogenital malformations. Eur. J. Med. Genet..

[9] Joodi M., Amerizadeh F., Hassanian S.M., Erfani M., Ghayour-Mobarhan M., Ferns G.A., Khazaei M., Avan A. (2019). The genetic factors contributing to hypospadias and their clinical utility in its diagnosis. J. Cell Physiol..

[10] Subramaniam R., Spinoit A.F., Hoebeke P. (2011). Hypospadias repair: An overview of the actual techniques. Semin. Plast. Surg..

[11] Askarpour S., Peyvasteh M., Mohamadi A., Khoshkhabar M. (2021). Comparative study of modifying meatal advancement glandular with release chordi versus Snodgrass surgical methods regarding the repair of distal hypospadias. World J. Plast. Surg..

[12] Asad S., Khan F.A., Ali S., Khan H., Rafaqat H., Din Khattak I.U. (2023). Snodgrass hypospadias repair at Ayub teaching hospital: An audit of complications and outcomes. J. Ayub Med. Coll. Abbottabad..

[13] Arshadi H., Sabetkish S., Kajbafzadeh A.M. (2017). Modified tubularized incised plate urethroplasty reduces the risk of fistula and meatal stenosis for proximal hypospadias: A report of 63 cases. Int. Urol. Nephrol..

[14] Moradi M., Kazemzadeh B., Hood B., Rezaee H., Kaseb K. (2016). Meatal mobilization and glanuloplasty: A viable option for coronal and glanular hypospadias repair. Urology.

[15] Seibold J., Boehmer A., Verger A., Merseburger A.S., Stenzl A., Sievert K.D. (2007). The meatal mobilization technique for coronal/subcoronal hypospadias repair. BJU Int..

[16] Seibold J., Werther M., Alloussi S., Gakis G., Schilling D., Colleselli D., Stenzl A., Schwentner C. (2010). Objective long-term evaluation after distal hypospadias repair using the meatal mobilization technique. Scand. J. Urol. Nephrol..

[17] Jukić M., Antišić J., Pogorelić Z. (2023). Incidence and causes of 30-day readmission rate from discharge as an indicator of quality care in pediatric surgery. Acta Chir. Belg..

[18] Jukić M., Biuk I., Pogorelić Z. (2022). The incidence and causes of unplanned reoperations as a quality indicator in pediatric surgery. Children.

[19] Snodgrass W.T. (2005). Snodgrass technique for hypospadias repair. BJU Int..

[20] Gozar H., Bara Z., Dicu E., Derzsi Z. (2023). Current perspectives in hypospadias research: A scoping review of articles published in 2021 (Review). Exp. Ther. Med..

[21] Zhang Y., Shen Z., Zhou X., Chi Z., Hong X., Huang Y., Huang H., Chen S., Lan K., Lin J. (2020). Comparison of meatal-based flap (Mathieu) and tubularized incised-plate (TIP) urethroplasties for primary distal hypospadias: A systematic review and meta-analysis. J. Pediatr. Surg..

[22] Winberg H., Arnbjörnsson E., Anderberg M., Stenström P. (2019). Postoperative outcomes in distal hypospadias: A meta-analysis of the Mathieu and tubularized incised plate repair methods for development of urethrocutaneous fistula and urethral stricture. Pediatr. Surg. Int..

[23] El-Helaly H.A., Youssof H.A., Ibrahim H.M., Aldaqadossi H.A., Abdalla O.M., Dogha M.M. (2022). Distal hypospadias repair: Comparative study between Snodgrass and transverse preputial onlay flap. J. Pediatr. Urol..

[24] Snodgrass W., Bush N. (2016). Primary hypospadias repair techniques: A review of the evidence. Urol. Ann..

[25] Soave A., Riechardt S., Engel O., Rink M., Fisch M. (2014). Complications of hypospadias repairs. Urologe A.

[26] Buisson P., Ricard J., Hamzy M., Pouzac M., Canarelli J.P. (2004). Evaluation of the results of the Snodgrass procedure in hypospadias surgery. Prog. Urol..

[27] Pfistermuller K.L., McArdle A.J., Cuckow P.M. (2015). Meta-analysis of complication rates of the tubularized incised plate (TIP) repair. J. Pediatr. Urol..

[28] Snodgrass W., Bush N.C. (2017). Re-operative urethroplasty after failed hypospadias repair: How prior surgery impacts risk for additional complications. J. Pediatr. Urol..

[29] Chukwubuike K.E., Obianyo N.E.N., Ekenze S.O., Ezomike U.O. (2019). Assessment of the effect of urethral plate width on outcome of hypospadias repair. J. Pediatr. Urol..

[30] Galal M., Taha D.E., Elabden K.Z., Nabeeh H., Abdelbaky T. (2021). The effect of pre-incision urethral plate width and glanular width on the outcome of tubularized incised urethral plate repair surgery in distal penile hypospadias, a prospective study. Urol. J..

[31] Bush N.C., Snodgrass W. (2017). Pre-incision urethral plate width does not impact short-term Tubularized Incised Plate urethroplasty outcomes. J. Pediatr. Urol..

[32] Akova F., Aydın E., Salabas E., Bilgili Z. (2022). Glans Diameter and Meatus localization are the sole predictors of primary distal hypospadias surgery complications: A multivariate analysis of single surgeon series. Cureus.

[33] Bush N.C., Villanueva C., Snodgrass W. (2015). Glans size is an independent risk factor for urethroplasty complications after hypospadias repair. J. Pediatr. Urol..

[34] Güler Y. (2020). TIPU outcomes for hypospadias treatment and predictive factors causing urethrocutaneous fistula and external urethral meatus stenosis in TIPU: Clinical study. Andrologia.

[35] Neheman A., Schwarztuch Gildor O., Shumaker A., Beberashvili I., Bar-Yosef Y., Arnon S., Zisman A., Stav K. (2024). Use of validated questionnaires to predict cosmetic outcomes of hypospadias repair. Children.

[36] Kumar A., Ram Dhayal I. (2022). A Comparative Study on the Outcomes of hypospadias surgery following early versus late bladder catheter removal. Cureus.

[37] Xu N., Xue X.Y., Wei Y., Li X.D., Zheng Q.S., Jiang T., Huang J.B. (2013). Outcome analysis of tubularized incised plate repair in hypospadias: Is a catheter necessary?. Urol. Int..

[38] Borkar N., Tiwari C., Mohanty D., Baruah T.D., Mohanty M., Sinha C.K. (2024). Post-urethroplasty complications in hypospadias repair: A systematic review and meta-analysis comparing polydioxanone and polyglactin sutures. World J. Pediatr. Surg..

[39] Pogorelić Z., Stričević L., Elezović Baloević S., Todorić J., Budimir D. (2024). Safety and effectiveness of triclosan-coated polydioxanone (PDS Plus) versus uncoated polydioxanone (PDS II) sutures for prevention of surgical site infection after hypospadias repair in children: A 10-year single center experience with 550 hypospadias. Biomedicines.

